# Using Geographic Information Systems and Spatial Analysis Methods to Assess Household Water Access and Sanitation Coverage in the SHINE Trial

**DOI:** 10.1093/cid/civ847

**Published:** 2015-11-11

**Authors:** Robert Ntozini, Sara J. Marks, Goldberg Mangwadu, Mduduzi N. N. Mbuya, Grace Gerema, Batsirai Mutasa, Timothy R. Julian, Kellogg J. Schwab, Jean H. Humphrey, Lindiwe I. Zungu

**Affiliations:** 1 Zvitambo Institute for Maternal and Child Health Research, Harare, Zimbabwe; 2 Swiss Federal Institute of Aquatic Science and Technology (Eawag), Duebendorf, Switzerland; 3 Ministry of Health and Child Care, Harare, Zimbabwe; 4 Center for Human Nutrition; 5 Department of Environmental Health Sciences, Johns Hopkins Bloomberg School of Public Health, Baltimore, Maryland; 6 Department of Health Studies, University of South Africa, Pretoria

**Keywords:** spatial analysis, water access, water coverage, geographic information systems, georeferenced dataset

## Abstract

Access to water and sanitation are important determinants of behavioral responses to hygiene and sanitation interventions. We estimated cluster-specific water access and sanitation coverage to inform a constrained randomization technique in the SHINE trial. Technicians and engineers inspected all public access water sources to ascertain seasonality, function, and geospatial coordinates. Households and water sources were mapped using open-source geospatial software. The distance from each household to the nearest perennial, functional, protected water source was calculated, and for each cluster, the median distance and the proportion of households within <500 m and >1500 m of such a water source. Cluster-specific sanitation coverage was ascertained using a random sample of 13 households per cluster. These parameters were included as covariates in randomization to optimize balance in water and sanitation access across treatment arms at the start of the trial. The observed high variability between clusters in both parameters suggests that constraining on these factors was needed to reduce risk of bias.

Achievement of the Millennium Development Goal (MDG) Target 7c (halving the proportion of people without sustainable access to safe drinking water) was an important milestone toward universal access to sufficient quantity and quality of water, a human right essential for human health and survival [1–4]. Target 7c defined “access” as the availability of at least 20 L of water per person per day from an improved source within 1 km [5].

However, the achievement of this target has been called an exaggeration of real progress because it does not account for several characteristics of water access proven to be important for human health: household-level walk time and distance to water, functionality and seasonality of water sources, per capita daily volume of water collected and used, and microbiological and chemical water quality at the point of use [6]. Additionally, achievement of MDG 7 at the country level often relied on combining national or subnational water-point surveys with population census data to calculate average or “theoretical” water coverage per capita. Because the density of both water points and populations may be highly variable across the regions assessed, such estimates often misrepresent access at the village and individual household level [7]. Accordingly, the post-2015 monitoring goals for “access to water” will likely include equity at the individual, household, or village level; microbial water quality; and infrastructure functionality [8]. In line with this shift, tools to measure and interpret these metrics will be required.

## BACKGROUND

The Sanitation Hygiene Infant Nutrition Efficacy (SHINE) trial is a cluster-randomized trial testing the independent and combined effects of improved water, sanitation, and hygiene (WASH) and improved infant feeding on child stunting and anemia at 18 months of age [9]. SHINE is motivated by the premise that environmental enteric dysfunction (EED) is a major underlying cause of stunting and anemia and that EED is primarily caused by infant ingestion of fecal microbes due to living in conditions of poor WASH. The SHINE WASH intervention includes provision of a ventilated improved pit latrine, Tippy Tap handwashing facilities and soap, a protected play space, point-of-use water chlorination solution, and behavior change communication to promote optimal WASH practices [10]. Notably, the intervention does not include a water access component. Because baseline water and sanitation access are both likely to affect uptake of the WASH interventions and trial outcomes through multiple pathways, we wanted to optimize balance of these parameters across treatment groups at the start of the trial. This required up-to-date cluster-specific estimates of water and sanitation coverage. The last comprehensive water and sanitation surveys in Zimbabwe had been conducted in 2004 and 2006, respectively [11]; neither provided data at the cluster level. Thus, we undertook the surveys described in this article to estimate cluster-specific water access and sanitation coverage to inform random allocation of clusters to treatment arms, thereby optimizing balance on these factors across arms.

## MATERIALS AND METHODS

### Digital Mapping of Study Site and Administrative Boundaries

The SHINE study site is comprised of the 2 adjacent Chirumanzu and Shurugwi districts, located in central Zimbabwe, with land areas of 4761 km^2^ and 3471 km^2^ and populations of 77570 and 80351, respectively [12] (see Figure 1 in Supplementary Appendix). Digital vector maps of administrative boundaries for the 2 districts were obtained from the Department of the Surveyor General [13] and Zimbabwe Statistics [14] as shape files [15], saved in the Universal Transverse Mercator projection datum WGS84 zone 35S [16].

### Data Collection

#### Mapping of Households

**Figure 1.**
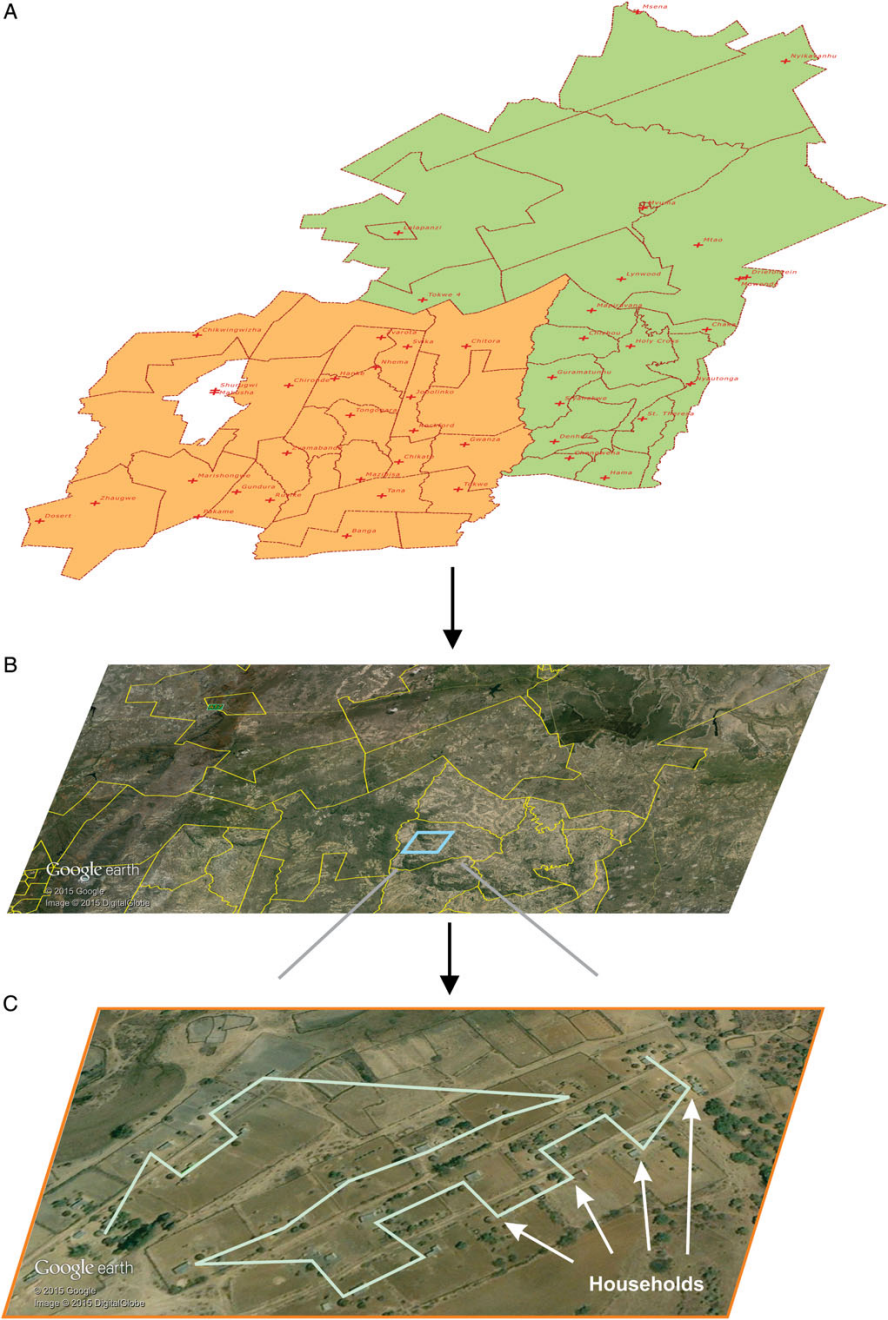
Households in the 2 districts were mapped by obtaining shape files for administrative boundaries (*A*), overlaying administrative boundaries on Google Earth imagery (*B*), and visually identifying and recording households using the ADD PATH tool in Google Earth (*C*). The jagged line in (*C*) is produced by the ADD PATH tool after sequentially selecting multiple households.

In November–December 2010, Google Earth (Google, Mountain View, California) was used to identify the coordinates (longitude, latitude, and elevation) of all households and key landmarks (roads, clinics, schools, rivers) and build a georeferenced household/key landmark dataset (Figure 1):

1. The digital district boundaries map was overlaid on Google Earth imagery to identify the study area. Within the study area, settlements identified as urban (having sewage connection and or piped water) or institutions were excluded from the study population and not mapped.
2. In Zimbabwe, rural households are generally comprised of a clearly demarcated plot of land with either one round or rectangular building or a grouping of several buildings each used for a different function (sleeping, cooking, storage, etc). Groups of households were mapped using the Google Earth “ADD PATH” tool by drawing a polyline where each vertex represented the center point of a household.
3. The polylines were exported from Google Earth as KML files (Open Geospatial Consortium 2012). Coordinates of each vertex (household) were extracted from the KML files into a dataset and assigned a unique identifier using a custom developed program. The georeferenced dataset of households and key landmarks was imported into QGIS version 2.6.1 (Open Source Geospatial Foundation, Arizona). The position accuracy of coordinates obtained by this method is comparable to that of land survey methods [17].

#### Mapping of Water Points

A desk study of available documents and organization documents (District Development Fund, Ministry of Health and Child Care [MoHCC], United Nations agencies, nongovernmental organizations, and district local authorities) was conducted to estimate the number and type of known water points in the study area to plan the survey. All water sources were physically inspected between January and June 2011. Four district water engineers assessed boreholes and deep wells while 22 MoHCC environmental health technicians (EHTs) assessed all other water points. At each water point, 2–4 key informants (pump minders, water committee members, village health workers, ward councilors, district maintenance officers, school heads, and village headmen) were interviewed. The data were collected electronically using personal digital assistants (model HP iPAQ 211, Hewlett-Packard, Palo Alto, California) paired with geographic positioning system (GPS) receivers (model BT-386i GPS Bluetooth, Globalsat, New Taipei City, Taiwan). The data acquisition program was developed using CyberTracker version 3.0 (CyberTracker, Cape Town, South Africa), which integrates GPS data with other captured data. Questionnaires were based on World Health Organization (WHO)/United Nations Children's Fund (UNICEF) guidelines [18] for water and sanitation household surveys, adapted in consultation with local water and sanitation experts, and pilot-tested in a neighboring rural district (Table 1 in Supplementary Appendix). The water-point dataset was exported from CyberTracker into a spreadsheet where each water point was assigned a unique identifier; thereafter, the georeferenced dataset was exported to QGIS for spatial analysis.

#### Formation of SHINE Clusters (Randomization Unit)

The study area was divided into 212 clusters, defined as the catchment area of 1–4 MoHCC village health workers (VHWs). Large-scale maps were produced by integrating the household/key landmark dataset with the district boundary dataset as geographic information system (GIS) layers. Each of the 360 VHWs circled the households they serve, creating a cluster boundary. Cluster-specific numbers of households, reproductive-aged women, and <2-year-old children were gathered from VHW registers. Cluster boundaries were finalized by grouping up to 4 if (1) they were working habitually as a team and (2) their catchment areas largely comprised households within close proximity (Figure 2 in Supplementary Appendix). A final cluster/household dataset was produced assigning a unique identifier to each cluster and a cluster identifier to each household.

#### Sanitation Survey

A random sample of 2756 households was selected, 13 from each of the 212 clusters, using the cluster/household dataset as the sampling frame. EHTs visited each selected household between January and March 2012, guided by the GPS location (Table 2 in Supplementary Appendix). If >1 household was found near the GPS location, one was chosen using a random number generator. Similar to the water-point survey, data were collected on personal digital assistants loaded with a sanitation survey based on WHO/UNICEF [18] guidance. Data were exported to QGIS for spatial analysis.

### Data Analysis

Spatial analysis and rendering of maps was performed using QGIS, and statistical analyses were performed using Stata/SE software version 13 (StataCorp, College Station, Texas).

#### Water Coverage

Water points were categorized using several definitions, but for the purposes of the constrained randomization, we considered only those that were fully functional, perennial, protected, and had unrestricted public access (defined as “optimal water source”). Nearest-neighbor analysis was used to calculate the shortest geographic distance from each household to the nearest water point; using the “DISTANCE MATRIX” function, the household/key landmark GIS layer was used as the input vector layer and the water-point GIS layer as the target in the computations, and results were exported for analyses using statistical software. Three cluster-specific metrics were used to constrain randomization of the clusters: median (interquartile range [IQR]) distance of all households to the nearest optimal water source, and proportion (95% confidence interval [CI]) of households <500 m or >1500 m from an improved water source. Heterogeneity of water access among the clusters was assessed using the intraclass correlation computed using the distance to water points [19]. To visualize coverage area of each water point, a buffer around each water point at 2 radiuses (500 m and 1500 m) was created and displayed on the maps.

#### Sanitation Coverage

Sanitation coverage was defined 3 ways for constrained randomization: the proportion of households per cluster with (1) any latrine, (2) a latrine less than half full, and (3) a latrine less than half full and a handwashing facility.

### Ethical Approval

The study was approved by the Medical Research Council of Zimbabwe, the Research Institute of McGill University Health Centre, and the Johns Hopkins Bloomberg School of Public Health. Verbal assent was obtained from a household adult prior to household latrine inspection.

## RESULTS

### Household Mapping

We identified 32 927 georeferenced households across the 2 districts: 16 313 in Chirumanzu and 16 614 in Shurugwi. Spatial distribution of the population (Figure 3 in Supplementary Appendix) illustrates 2 distinct settlement patterns: (1) dense settlements in older communal areas established during the colonial era, and (2) clustered settlements in newer resettlement areas established following independence in 1980. Applying average household size in each district according to the 2012 census, 32 927 households correspond to an estimated population of 133 484 (84.5% of the population reported in the 2012 census for both districts) [12]. The remaining 15.5% of the population is likely accounted for by exclusion of urban and growth point settlements (also excluded from the SHINE trial), and areas that did not have clear satellite imagery to identify households.

### Water-Point Mapping

A total of 8388 water points were mapped and characterized; this was approximately 50% more than those initially identified by key informants before the beginning of the survey. Our method of sourcing all water-point information from 2–4 key informants from the community likely yielded a complete count (census) of water points. Of the water points, 75% were shallow wells. Most water points had a bucket water removal system, were protected, and were publicly accessible, but only 60% were perennial (Table 3 in Supplementary Appendix).The spatial distribution of the 7906 (97.2%) functional water points is shown in Figure 4 of the Supplementary Appendix.

### Water Coverage

**Figure 2.**
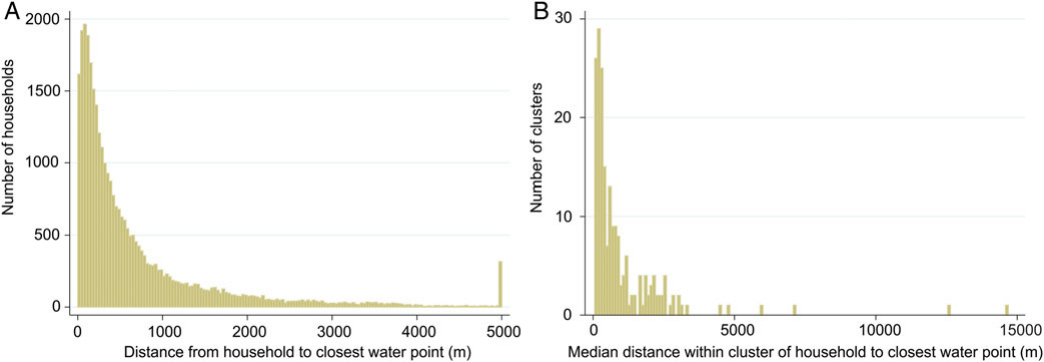
Distribution of distance from household to closest water point across the Sanitation Hygiene Infant Nutrition Efficacy study area (*A*), and the median distance within clusters (*B*).

**Figure 3.**
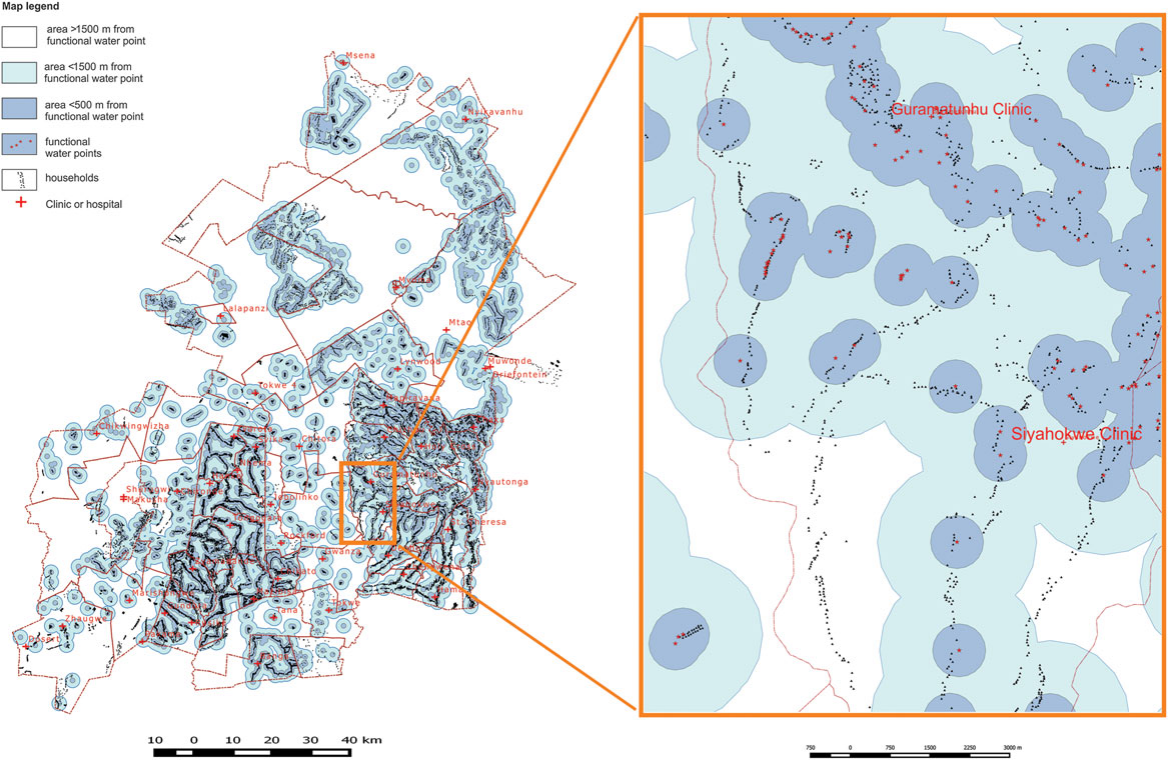
Spatial water coverage by functional, protected, perennial, and unrestricted water points across the Sanitation Hygiene Infant Nutrition Efficacy study area. The insert shows households that fall into the 3 regions: within <500 m, <1500 m, and >1500 m of a water point.

Across the SHINE study area, the median distance between households and any source of water was 182.8 m (IQR, 74.7–404.7 m), and 31.4%, 78.6%, and 94.3% of households were within 100 m, 500 m, and 1500 m, respectively. When considering only “optimal” water points, these respective values were 360.9 m (IQR, 146.4–848.4 m), and 16.5%, 58.2%, and 84.3%. Further estimates based on various definitions combining functionality, restricted use, protection, and seasonality are summarized in Table 1. Thus, access to water was highly variable and skewed to high across the 33 000 households (Figure 2*A*). Access to water at the cluster level was similarly variable (intraclass correlation for distance to water point was 0.738; *P* < .0001) and skewed (Figure 2*B*). Across the 212 clusters, the median distance of households within a cluster to optimal water ranged from 34.0 m (IQR, 18.2–68.1 m) for the cluster with best water access to 5556 m (IQR, 4804–6964 m) for the cluster with the worst water access; the proportion of clusters with a median distance to optimal water <100 m, <500 m, and >1500 m was 19.0%, 80.5%, and 93.5%, respectively. Maps depicting spatial water coverage were created (Figure 3).

**Table 1.** Summary of Water Access Using Various Definitions of Water

Water Access Measure	Water Definition				
	Any WP (n = 8388)	Functional WP (n = 7906)	Functional, and Nonrestricted WP (n = 7110)	Functional, Nonrestricted, and Perennial WP (n = 4151)	Functional, Nonrestricted, Perennial, and Protected WP (n = 3034)
HH < 100 m, % (95% CI)	31.4 (30.9–31.9)	31.3 (30.8–31.8)	29.4 (28.9–29.9)	19.1 (18.7–19.5)	16.5 (16.1–16.9)
HH < 500 m, % (95% CI)	78.6 (78.1–79.0)	75.5 (75.1–76.0)	74.0 (73.5–74.5)	66.2 (65.6–66.7)	58.2 (57.7–58.7)
HH < 1000 m, % (95% CI)	91.0 (90.7–91.4)	88.7 (88.4–89.1)	87.8 (87.4–88.2)	83.8 (83.4–84.2)	76.6 (76.2–77.1)
HH < 1500 m, % (95% CI)	94.3 (94.1–94.6)	93.9 (93.6–94.1)	93.3 (93.0–93.6)	90.6 (90.3–91.0)	84.3 (83.9–84.7)
Mean (SD)	335.7 (493.2)	492.3 (1092.0)	514.8 (1102.0)	635.2 (1152.8)	730.5 (1124.5)
Median (IQR)	182.8 (74.7–404.7)	197.7 (77.1–488.4)	211.2 (84.2–522.1)	305.0 (130.3–672.7)	360.9 (146.4–848.4)

Abbreviations: CI, confidence interval; HH, household; IQR, interquartile range; SD, standard deviation; WP, water point.

### Seasonal Variation in Water Coverage

**Figure 4.**
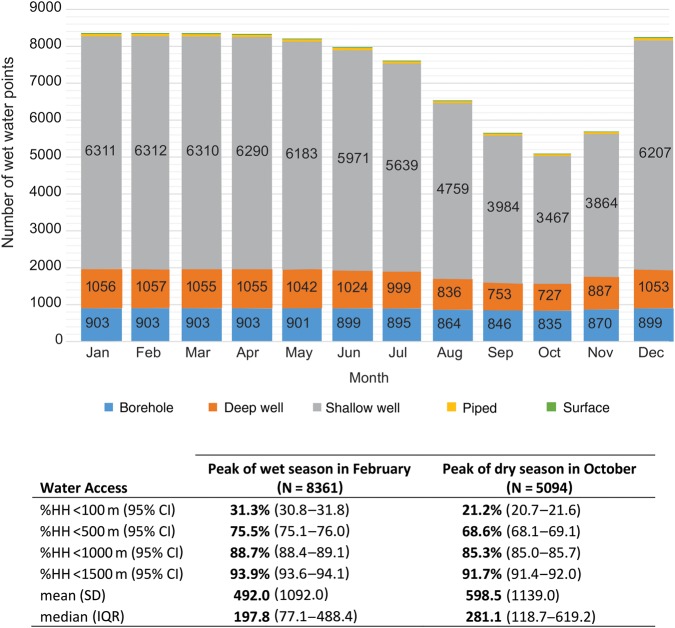
Seasonal variation of available functional water points showing water coverage during the wet and dry seasons. Abbreviations: CI, confidence interval; HH, household; IQR, interquartile range; SD, standard deviation.

Seasonality was the most prevalent factor limiting water sources from being considered optimal. The number of water points with water ranged from 5094 in October to 8361 in February, bracketing the annual rainy season. The proportion of households with access to a water point with water within 100 m, <500 m, and >1500 m was 31.3% (95% CI, 30.8%–31.8%), 75.5% (95% CI, 75.1%–76.0%), and 93.9% (95% CI, 93.6%–94.1%), respectively, in February. In October, these relative values were 21.2% (95% CI, 20.7%–21.6%), 68.6% (95% CI, 68.1%–69.1%), and 91.7% (95% CI, 91.4%–92.0%), respectively. The predominant water-point type for households with water within <100 m was shallow wells, which were also the type most affected by seasonality, dropping by 45% between February and October. Figure 4 shows the trend of available water sources over a calendar year.

### Sanitation Coverage

A total of 2560 households (92.9% response rate) were assessed for sanitation coverage. Of these, 1148 (44.8%) had a latrine of any type, but 373 (32.5%) of these were full and no longer in use, resulting in a net sanitation coverage of 30.3% (775/2560). Moreover, of these 775, only 133 (12%) had a ventilation pipe fitted that passed the ventilation test and had a fly screen. Only 75 latrines had handwashing facilities, and only 20 had water and 1 had soap. In 9 (0.8%) latrines, there was evidence the latrine was being used for storage or for a purpose other than urination or defecation. Sanitation coverage at the cluster level ranged widely; in the cluster with the poorest and best sanitation coverage, 7.7% and 92.3%, respectively, of the households had a latrine of any type.

## DISCUSSION

**Figure 5.**
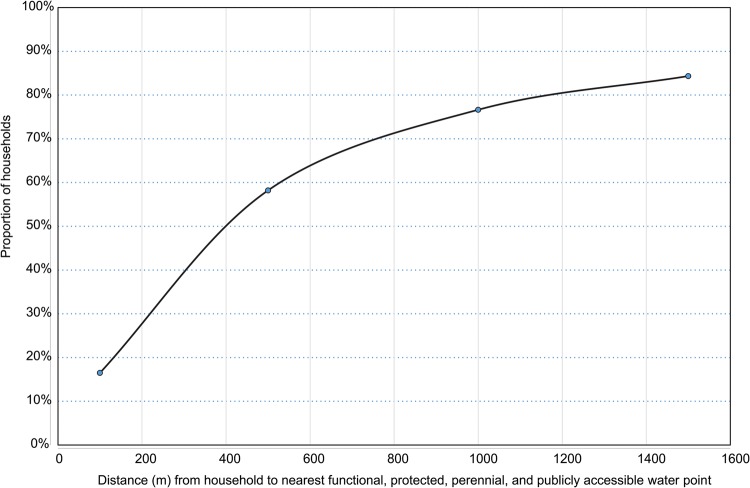
The proportion of the population covered by functional, protected, perennial, and unrestricted water points as distance from the water point changes.

The primary purpose of this study was to constrain randomized allocation of clusters to treatment groups to ensure balance in water access and sanitation coverage across treatment groups. The high variability between clusters in both water access (Figure 5) and sanitation suggests that constraining on these factors was prudent in reducing risk of bias from imbalances across groups.

For most households in the SHINE study area, access to water represents a daily hardship: 16.5%, 58.2%, and 15.5% of the 32 927 households were <100 m, <500 m, and >1500 m, respectively, from the nearest optimal (ie, functioning, protected, publicly accessible, perennial) source of water; the median household was 361 m, or an approximately 11-minute one-way walk time, from such a water point. The Water Use Plateau describes the relationship between distance traveled to a source and volume of water consumed, with a rapid decline in consumption when distance exceeds 100 m (approximately a 3-minute one-way walk time) [20]. Bartram et al [7] estimate that where a water source is >1000 m (>15-minute one-way walk time), water volumes accessed are too little to support hydration or hygiene requirements and are thus associated with higher risk of adverse health consequences. Furthermore, they report that a basic water service level (within 100–1000 m) supports hydration but not optimal hygiene and is similarly associated with higher risk of poor health. Only when water is provided on-plot (within 100 m or <3 minutes) did consumption reach levels associated with lower risk of adverse health consequences.

In a large-scale empirical study, Pickering and Davis [21] used Demographic Health Survey data from 26 countries to model the effect of water collection time on child health. Each 15-minute reduction in one-way walk time to water was associated with substantial reductions in child morbidity and mortality, and, of most relevance to SHINE, increased linear growth. Consistent with previous studies, the child health effect of water access was modified by sanitation: Health impacts were greater among children in households with sanitation compared with those practicing open defecation. Moreover, even when households with an on-plot water source were excluded, the walk-time health effect decreased only slightly, suggesting that relative distance to off-site water remains strongly related to child health [21]. The authors suggested that the observed adverse effects of longer walk times might be mediated by reduced volumes available for hygiene, prolonged drinking water storage, less capacity for home gardening, and less maternal time for child care and income generation [21, 22].

Seasonality in water access was also highly variable; from the end of the wet season to the end of the dry season, the number of water points with water declined by 39%. Thus, when infants age into particular vulnerable periods season may confound outcomes in the trial and/or modify intervention effects.

Less than half of the households (45%) had a latrine and 34% of these were full and no longer in use, such that net sanitation coverage was 30%. However, virtually none of the latrines were being used for any purpose other than urination, defecation, and bathing. This is consistent with previous formative studies that revealed that having a latrine and using it for sanitation is highly valued in this population [10].

This study makes several methodological contributions to the growing area of water-point and sanitation service mapping. First, using a set of free and low-cost tools, we developed an integrated system for estimating the geospatial distribution of households, water sources, and sanitation facilities and computation of distance-based metrics for physical access to water resources. Several other open-source platforms are available to map water points (eg, Water Point Mapper, Field Level Operations Watch [FLOW], and WASHTech), but none has the technical capacity to integrate household coordinates. These other platforms rely on census data to gauge access to water and sanitation, and the averaged estimates lack the necessary resolution to reveal spatial trends that are critical for planning future investments. The household-based strategy represents a particular improvement where populations are dispersed unequally and water is scarce.

Second, this study extends the literature on water and sanitation mapping by demonstrating how mapping efforts can be fully integrated within existing institutions. Data collection at the household and community level was accomplished through close collaboration with personnel in the MoHCC. EHTs' routine visits to rural wards provided the access to and knowledge of rural wards throughout the 2 study districts. Service providers are increasingly recognizing the importance of obtaining accurate and accessible geospatial data for strategic planning and monitoring of water and sanitation programs [23, 24].

Third, the approach taken in this study may be especially salient in the post-2015 development period, when definitions of access are likely to include aspects of equity, microbial water quality, and infrastructure functionality [8].

Fourth, this methodology reveals patterns and trends that may be obscured in regional- or national-level assessments. For example, it is possible to use spatial statistics to identify clusters of households where sanitation access is limited or to investigate key covariates of water-point functionality, such as proximity to roads or access to spare parts (for repair and maintenance).

**Figure 6.**
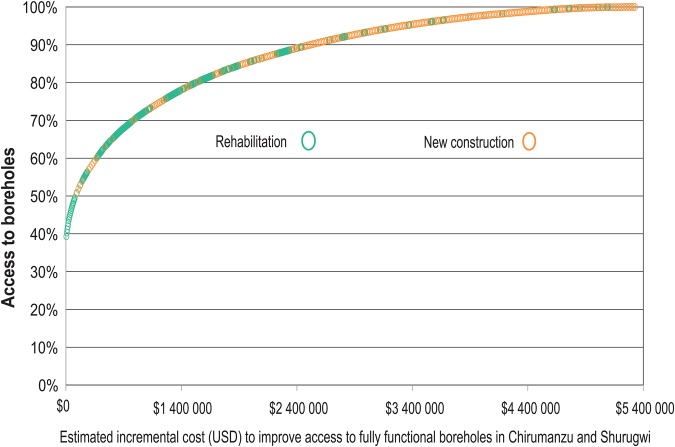
Modeling the cost of improving water coverage by mixing rehabilitation of broken boreholes and drilling for replacement boreholes based on fixed cost estimates for replacement and rehabilitation.

Finally, household-based geospatial data on water-point infrastructure characteristics can be used to plan strategic investments in their maintenance and upgrades. For example, using the functionality and location for each water point, we modeled the incremental proportion of the population with access to water for each additional dollar spent (Figure 6). This model reveals the most cost-effective mix of repair work and new construction to gain the greatest increases in the proportion of the population with water access.

There were 2 main limitations to our study. Google Earth may miss some households when the program is out of date, cloud coverage obscures imagery, or 2 households are close together and appear as one. These limitations can be reduced by requesting EHTs to “ground-truth” data during their routine visits to communities, and override household geospatial data. EHTs can also be trained to routinely update the database with changes in water-point functionality, access, or protection. A second limitation is that the methodology depends on a geographic distance–based measure for access which fails to account for topography or social drivers of a household's access to water.

## CONCLUSIONS

In this context, the observed high variability in water and sanitation access between clusters confirms that the SHINE approach of constraining on these factors during cluster randomization was needed to reduce risk of bias. Linking the water-point data with SHINE participants will help to understand heterogeneity in WASH practices and child health outcomes [25]. The methods described in this article also have wider applications and utility, as they can be used to provide accurate estimates of geospatial distribution of water and sanitation facilities and computation of distance-based metrics for physical access to water resources. These metrics are essential for the epidemiological study of child health and also in planning, monitoring, and evaluating water and sanitation supply programs.

## Supplementary Data

Supplementary materials are available at *Clinical Infectious Diseases* online (http://cid.oxfordjournals.org). Supplementary materials consist of data provided by the author that are published to benefit the reader. The posted materials are not copyedited. The contents of all supplementary data are the sole responsibility of the authors. Questions or messages regarding errors should be addressed to the author.

Supplementary Data
