# Supplementary material for: Using Geographic Information Systems and Spatial Analysis Methods to Assess Household Water Access and Sanitation Coverage in the SHINE Trial
Source: Clin Infect Dis. 2015 Nov 11;61(Suppl 7):S716–25. doi: 10.1093/cid/civ847 (PMC4657592; doi:10.1093/cid/civ847)
Supplement: Supplementary Data [file supp_61_suppl-7_S716__index.html]

Supplementary Data 

# Using Geographic Information Systems and Spatial Analysis Methods to Assess Household Water Access and Sanitation Coverage in the SHINE Trial

## Supplementary Data

Supplementary Data

- Supplementary Data - Docx file
- Supplementary Figure 1 - tif file
- Supplementary Figure 2 - tif file
- Supplementary Figure 3 - tif file
- Supplementary Figure 4 - tif file
- Supplementary appendix - docx file
