# Supplementary material for: Using Geographic Information Systems and Spatial Analysis Methods to Assess Household Water Access and Sanitation Coverage in the SHINE Trial
Source: Clin Infect Dis. 2015 Nov 11;61(Suppl 7):S716–25. doi: 10.1093/cid/civ847 (PMC4657592; doi:10.1093/cid/civ847)
Supplement: Supplementary Data [file supp_civ847_civ847supp.docx]

**Supplementary figure legends**

**Supplementary figure 1:** Map of the SHINE study districts in Zimbabwe

**Supplementary figure 2:** Creating SHINE clusters: (A) VHW markings of catchment areas on large scale maps showing location of households. (B) Map showing finalized cluster boundaries, the colors depict SHINE treatment arms

**Supplementary figure 3:** Distribution of the mapped households across SHINE study showing two distinct settlement patterns (A) newer villagized resettlement areas and (B) older communal areas

**Supplementary figure 4:** Distribution of water point across SHINE study area. (A) Shows an area with a high water table where the predominant water points area shallow wells and almost all household have their own private well. (B) Shows a dry area where the only water points are boreholes and households walk long distances to water
