# Supplementary material for: Using Geographic Information Systems and Spatial Analysis Methods to Assess Household Water Access and Sanitation Coverage in the SHINE Trial
Source: Clin Infect Dis. 2015 Nov 11;61(Suppl 7):S716–25. doi: 10.1093/cid/civ847 (PMC4657592; doi:10.1093/cid/civ847)
Supplement: Supplementary Data [file supp_civ847_civ847supp_appendix.docx]

**Appendix 4: Table 1.** Characteristics of water point assessed during the water point survey.

| ***Survey Category*** | ***Possible answers*** | ***Definition*** |
| --- | --- | --- |
| Location | GPS coordinates | Capture GPS coordinates of location |
| Water point type | Deep borehole  Protected well  Unprotected well  Tap stand  Unprotected spring, ….stream, or river  Dam  Other | A water point is categorized as *improved* based on WHO/UNICEF Joint Monitoring Program guidelines: By nature of its construction, the water source is expected to be protected from outside contamination, especially fecal contamination. These include deep borehole, protected well, tap stand, bottled water, or rainwater harvesting. |
| Institutional arrangement | Communal  Institutional  Institutional/communal  Homestead | ‘Communal’ includes public water points with or without restrictions on use; ‘institutional’ includes clinics, schools, etc.; ‘homestead’ refers to private water points. |
| Protection | Full  Partial  None  N/A | Unique characterization based on the water point type, with the following general guidelines:  Full = little to no chance for re-contamination due to protective fence, apron, drain, adequate lid; partial = possibility of re-contamination, some aspect of fence, apron, or lid is missing; none = recontamination likely, no protection present; N/A = water point cannot be protected (e.g., river). |
| Seasonality | Perennial  Year round | ‘Perennial’ refers to water sources that are typically dry at any point during the year; ‘year round’ refers to sources that provide water year round |
| Number of users | (number) | Number of people who used the water source regularly |
| Abstraction unit | Type A bush pump  Type B bush pump  Diesel/electric pump  Bucket with windlass  Bucket with rope  Bucket only  Rope/washer pump  None  Other | Based on Government of Zimbabwe design standards for domestic water supply infrastructure in rural areas |
| Operational status | Full  Partial  Limited  None | For hand-pumps, ‘full’ is <20 strokes/20L; ‘partial’ is 20-40 strokes/20L; ‘limited’ is 40-100 strokes/20L; ‘none’ is >100 strokes/20 L. For motorized pumps, ‘full’ is >1 L/s; ‘partial’ is 0.5-0.9 L/s; ‘limited’ is 0.1-0.5 L/s; ‘none’ is <0.1 L/s. For buckets, ‘full’ is <3 min/20L; ‘partial’ is 3-10 min/20L; ‘poor’ is 10-30 min/20L. If no abstraction unit, no operational test was performed. |
| Reason for being partial/non-operational  (check all that apply) | Mechanical  Water source issue  Social issue | Mechanical issues include broken or missing components such as the pump or abstraction unit. Water source issues include problems with the aquifer, casing/lining, the sanitary seal, or the apron. Social/maintenance issues include evidence of vandalism, lack of spare parts, lack of skills locally, or restricted access. |
| Repairs required | Labor estimate (hours)  Materials estimate (US$) | Based on visiting engineers informed estimates. |

**Appendix 4: Table 2.** Characteristics of sanitation facilities assessed during the sanitation field survey.

| ***Survey question*** | ***Possible answers*** |
| --- | --- |
| **Facility location** | [GPS coordinates] |
| **Facility design** | Blair Ventilated Improved Pit (BVIP) |
|  | Other design |
| **Pit status** | Full |
|  | About half full |
|  | About empty |
| **Has ventilation pipe?** | Y/N |
| **Has fly screen?** | Y/N |
| **Passed smoke test?** | Y/N |
| **Other uses?** | What other uses latrine is used for |
| **Users?** | [number] |

**Appendix 4: Table 3:** Characteristics of the water points

|  |  | **Type of water source** | | | | | | | | | | | |
| --- | --- | --- | --- | --- | --- | --- | --- | --- | --- | --- | --- | --- | --- |
|  |  | BOREHOLE (N=922) | | DEEP WELL (N=1058) | | SHALLOW WELL (N=6318) | | PIPED  (N=63) | | SURFACE (N=27) | | TOTAL (N=8388) | |
| Type of lifting device n(%) | 1. pump | 882 | (95.7%) | 255 | (24.1%) | 39 | (0.6%) | 7 | (11.1%) | 2 | (7.4%) | 1185 | (14.1%) |
|  | 1. bucket | 5 | (0.5%) | 792 | (74.9%) | 6255 | (99.0%) | 50 | (79.4%) | 19 | (70.4%) | 7121 | (84.9%) |
|  | 1. other | 35 | (3.8%) | 11 | (1.0%) | 24 | (0.4%) | 6 | (9.5%) | 6 | (22.2%) | 82 | (1.0%) |
| Protected n(%) |  | 822 | (89.2%) | 1049 | (99.2%) | 4009 | (63.5%) | 30 | (47.6%) | 10 | (37.0%) | 5920 | (70.6%) |
| Perennial n(%) |  | 832 | (90.2%) | 716 | (67.7%) | 3382 | (53.5%) | 47 | (74.6%) | 17 | (63.0%) | 4994 | (59.5%) |
| Functional n(%) |  | 567 | (77.9%) | 985 | (96.9%) | 6270 | (99.5%) | 61 | (99.9%) | 23 | (99.9%) | 7906 | (97.2%) |
| Not restricted n(%) | | 112 | (12.2%) | 154 | (14.6%) | 581 | (9.2%) | 9 | (14.3%) | 5 | (18.5%) | 861 | (10.3%) |
